# A rapid review of best practices in the development of risk registers for public health emergency management

**DOI:** 10.3389/fpubh.2023.1200438

**Published:** 2023-11-30

**Authors:** Danylo Kostirko, Jiawei Zhao, Melissa Lavigne, Benoit Hermant, Liam Totten

**Affiliations:** Risk and Capability Assessment Unit, Public Health Agency of Canada, Ottawa, ON, Canada

**Keywords:** risk register, prioritization, risk assessment, risk management, emergency management, health security, public health, vulnerability

## Abstract

**Introduction:**

Public health organizations (PHO) must prepare to respond to a range of emergencies. This represents an ongoing challenge in an increasingly connected world, where the scope, complexity, and diversity of public health threats (PHT) have expanded, as exemplified by the COVID-19 pandemic. Risk registers (RR) offer a framework for identifying and managing threats, which can be employed by PHOs to better identify and characterize health threats. The aim of this review is to establish best practices (BP) for the development of RRs within Public Health Emergency Management (PHEM).

**Methods:**

In partnership with a librarian from Health Canada (HC), and guided by the Cochrane Rapid Review Guideline, journal articles were retrieved through MEDLINE, and a comprehensive search strategy was applied to obtain grey literature through various databases. Articles were limited to those that met the following criteria: published on or after January 1, 2010, published in the English language and published within an Organisation for Economic Co-operation and Development setting.

**Results:**

57 articles were included for synthesis. 41 papers specifically discussed the design of RRs. The review identified several guidelines to establish RRs in PHEM, including forward-looking, multidisciplinary, transparent, fit-for-purpose, and utilizing a systems approach to analyze and prioritize threats. Expert consultations, literature reviews, and prioritization methods such as multi-criteria-decision-analysis (MCDA) are often used to support the development of RRs. A minimum five-year-outlook is applied to assess PHTs, which are revisited yearly, and iteratively revised as new knowledge arises.

**Discussion:**

Based upon this review, RRs offer a systems approach to PHEM that can be expanded to facilitate the analysis of disparate threats. These approaches should factor in the multidimensionality of threats, need for multi-sectoral inputs, and use of vulnerability analyses that consider inherent drivers. Further research is needed to understand how drivers modify threats. The BPs and recommendations highlighted in our research can be adopted in the practice of PHEM to characterize the public health (PH) risk environment at a given point in time and support PHOs policy and decision-making.

## 1 Introduction

Recently emerged and re-emerging public health threats (PHT) have had devastating global economic and social impacts. In Canada, managing PHTs has become more difficult due to their increasing complexity in origin, characteristics, and influence on societal changes (1, 2). Moreover, emerging and re-emerging infectious diseases, as well as natural disasters such as floods, are increasing in frequency and intensity (2, 3). In fact, in the last two decades alone, at least one new emerging disease has been identified every year. These challenges highlight a need to develop forward-looking approaches that also take into consideration the disparate and complex nature of PHTs (1, 2, 4).

Canada currently lacks a holistic and integrated prioritization approach for PHTs (3). These PHTs can be characterized as either chemical, biological, radiological or nuclear agents and are often prioritized on partially subjective activities such as horizon scanning (3, 5–8). Presently, PH programs are also disjointed, and expertise segmented (8). As a result, under existing conditions, it is difficult to prioritize a diverse set of PHTs without being influenced by biases that arise from professional and political foci, interests, priorities or motives (3, 5, 6, 8). Similarly, PH preparedness and planning is currently targeted at known, emerging, and/or re-emerging infectious diseases (9). These are significant problems because PHTs do not care for political opinion, nor do they remain static over time. Instead, the likelihood and resulting impacts of pathogens shift in relation to changing drivers such as climate change, global travel and trade, immigration patterns, and urbanization (3, 9, 10) Lastly, infrequent and high-impact threats will inevitably be missed if decision makers fail to look past political terms and ignore the driving forces of disease. Thus, it is essential to resolve these issues and implement appropriate solutions for sustainable and effective decisions to be made (9).

The 2021 report of the Auditor General of Canada, *Pandemic Preparedness, Surveillance, and Border Control Measures*, highlighted that “decision makers need credible and timely risk assessments to guide effective responses” (4). A RR is a structured document that identifies and records potential risks, their impacts, and associated plans. It serves as a central repository for managing and mitigating risks and can help organizations prioritize threats. However, currently, methods, research, and standards to guide threat prioritization approaches within PH are lacking (11, 12). Only a few publications describe the methodologies sufficiently to allow for reproducibility or adaptation in other settings (12). The volume of publications in terms of actual prioritization results is also inadequate (12). Although PHOs have worked with processes to rank and prioritize PHTs, the efficacy of these approaches has yet to be systematically analyzed. Consequently, different organizations have adopted or proposed varying methods to prioritize PHTs.

Considering the finite nature of organizational resources, there is a pressing need to refine PH risk assessment methodologies to guarantee effective and efficient resource allocation. This review endeavors to elucidate BPs for threat prioritization within the realm of PHEM. By identifying the BPs, resources can be optimally directed towards the most pressing challenges, ultimately mitigating the economic and social repercussions stemming from the emergence or re-emergence of PHTs.

## 2 Methods

### 2.1 Article identification

In partnership with a librarian from HC, and guided by the Cochrane Rapid Review Guideline, articles were retrieved from MEDLINE, and a broad search strategy was applied to obtain grey literature from various databases. Our review focused on the theme of RRs as PH decision-making tools, and investigated the concepts of risk analysis, prioritization, identification, classification, and characterization. An extensive list of keywords was formulated to ensure that we comprehensively captured the literature. The list of keywords utilized for this review can be found in Appendix A, and the questions which guided our research are listed in Appendix B.

We limited our search to articles published in the English language and to literature published on or after January 1, 2010. We selected January 1, 2010, as our inclusion threshold to ensure that the literature we reviewed captured the guidance provided by the International Organization for Standardization 31,000 which was first published in 2009 and later updated in 2018. To make our findings relevant to the practice of PHEM within Canada, we only included articles published in the context of an Organisation for Economic Co-operation and Development (OECD) member country. Literature that was focused on the clinical level or articles with a specific use case (i.e., wastewater management) were excluded. A subsequent supplementary grey literature search was conducted by the same HC librarian who supported the development of the primary search strategy. Examples of grey literature sources searched include Google Scholar, the World Health Organization website, the European Center for Disease Prevention and Control website, and various other governmental and supra-national websites. Additional research was obtained through consultations with the Risk and Capability Assessment Unit, and reference searching.

### 2.2 Article screening

Our initial search strategy identified 1,171 records for article screening. Before title, and abstract screening could begin, records from the database search were uploaded to DistillerSR. Three duplicates were found using the detection tool and removed. The researchers then devised the inclusion, and exclusion criteria for title, and abstract screening.

Two reviewers, DK, and JZ, followed a modified version of the Cochrane Rapid Review protocols. Specifically, to accelerate article screening, a single-stage screening method was implemented where titles and abstracts were screened together, and followed by a second reviewer verification. Since titles and abstracts were screened together, the researchers set broad inclusion criteria to minimize the risk of excluding relevant research. Specifically, to be included in full-text screening, the article had to have relevance to RRs or their components: threat identification, analysis, and characterization. The researchers also included articles related to PH decision tools, processes or standards.

The reviewers allocated 30 training articles for title, and abstract screening and 5 for full-text screening. Disagreements for the inclusion or exclusion of an article were resolved through discussion, and a third reviewer ML was brought in for unresolved articles. Once the reviewers established confidence, and consensus, the two reviewers proceeded to independently screen articles. In the title and abstract screening, 1,041 articles were excluded, leaving 127 articles for full-text screening. Articles reviewed during full-text screening were excluded if they were focused at the clinical level, a special use case, or a commentary, editorial, narrative or opinion paper. In the end, 70 articles were excluded during full-text screening- leaving 57 articles for data extraction.

### 2.3 Data extraction

Data extraction fields included; study objectives; intended use; intended end user(s); PHTs, and their rank; PH drivers; findings related to RR design; methods for stakeholder engagement; timeline for revisions; threat identification; criteria to include or exclude a threat; methods to prioritize threats; methods to rank threats; criteria to categorize threats; uncertainty; vulnerability; any stated benefits; limitations; barriers to implementation; suggestions for implementation; conclusions; most significant findings; BPs, and any recommendations set by the author.

These questions were transposed to a data extraction form on DistillerSR, which the two reviewers DK, and JZ subsequently used for data extraction. Articles for data extraction were split between both researchers. To ensure data extraction was conducted accurately, and comprehensively, a process for quality control followed afterwards. Specifically, if any articles were technical, highly complex or information-dense, they were flagged for a second round of review. Moreover, the secondary researcher reviewed all the data extraction forms, and literature to ensure the quality of the data extraction process.

Literature was marked for further analysis if it directly discussed a RRs design, components, criteria to include and exclude threats, criteria to prioritize threats, or methods for stakeholder engagement. Likewise, studies with a national focus were flagged for further review as such studies aligned with our research goals and intended audience.

### 2.4 Synthesis of studies

The data extraction form from DistillerSR was transposed to Microsoft Excel, and quantitative, and qualitative findings were analyzed through thematic analysis. DistillerSR is a systematic review software designed to streamline the process of literature screening and data extraction for research studies. Thematic analysis involved content analysis of RR data and identification of recurring patterns and categories. We employed thematic coding (e.g., fit-for-purpose, holistic, multi-disciplinary etc.) to uncover key themes, providing insights into the BPs for developing RRs for PHEM. Thematic analysis on RR BPs and principles, limitations, and benefits were based on qualitative data extracted from the discussion sections of all papers.

## 3 Results

Of the 57 articles analyzed, (48%, *n* = 27) were intended for government end-users (e.g., policy, decision-makers and PHEM). An overwhelming majority of these articles were directed at national level governments (44%, *n* = 25). two articles were targeted at the regional level, and 1 was targeted towards the local level, yet the 2 articles were intended for regional level government.

**Figure 1:**
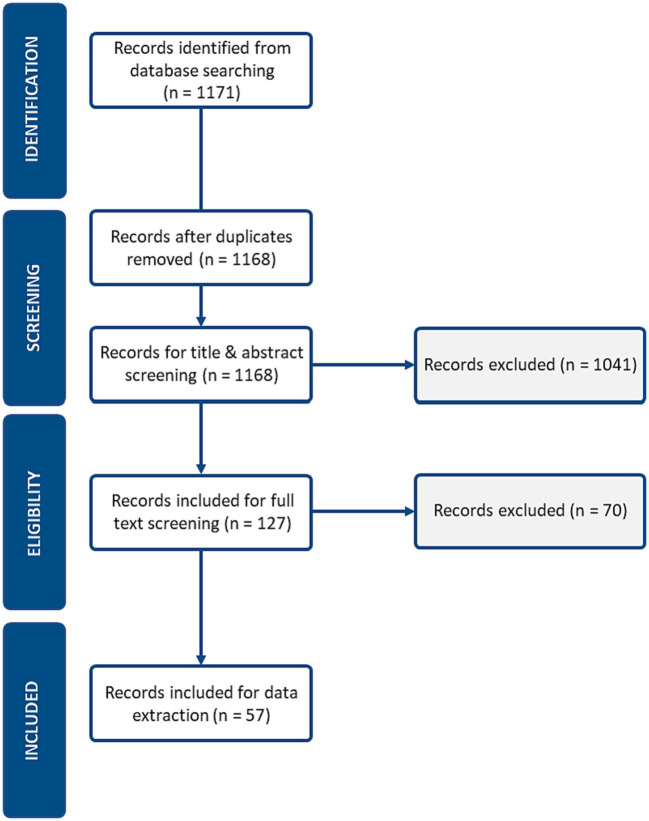
PRISMA flow diagram.

A PRISMA flowchart of the literature search is shown in Figure 1.

During the title and abstract screening phase, Cohen’s Kappa was utilized to assess inter-rater agreement. Cohen’s kappa is a statistical coefficient that measures the agreement between two raters for categorical data, accounting for chance. A score of 0.54 was obtained and the Cohen’s Kappa interpretation of 0.54 is “moderate.” This score reflected only the journal articles retrieved from the primary search strategy and from MEDLINE and did not include grey literature, and articles obtained from the supplementary search. A Kappa score was not calculated for full-text screening as all articles were discussed between both reviewers, and articles were only included for synthesis if consensus was reached.

### 3.1 General context

An analysis of the article’s objectives elucidated that most articles sought to enhance PHEM practice (13–15). Specifically, the objectives for the majority of literature reviewed could be categorized as either method development, enhancement or testing. Secondary objectives included improving the efficiency, effectiveness, and sustainability of PHEM decisions (1, 16, 17). Other objectives highlighted in the research include reducing morbidity, and mortality as consequence of threats emerging, and preserving PH resources (18). In all references included, two primary methods were utilized to achieve these objectives: literature reviews, and method testing.

### 3.2 Risk register: principles

Many studies highlighted key guidelines for RRs within PHEM. First, RRs should be fit-for-purpose: ensuring that the methods, processes, structure, and criteria are appropriate to the problem at hand, the organization’s objective(s), management needs, the purpose of the prioritization exercise, and considerate of regulatory, and non-regulatory laws, agreements, mandates, and policies (19–22). Second, RRs should be iterative, recursive, and flexible: ensuring that they are structured to support cyclical, and cycle improvements, adapted to feedback, new knowledge, and changing circumstances, and organizational priorities (13, 21, 23–25). Third, RRs should be holistic, and grounded in a systems level understanding so that all relevant variables are assessed, and the multi-disciplinary nature of PHEM is respected (12, 26). Fourth, the principles of transparency, consistency, and repeatability are critical, as findings should be evidence-based, valid, and auditable (13, 21, 24). Lastly, interoperability is essential as threat prioritization exercises inform other aspects of PHEM such as capability development (13, 21, 27, 28).

### 3.3 Components and process

**Figure 2:**
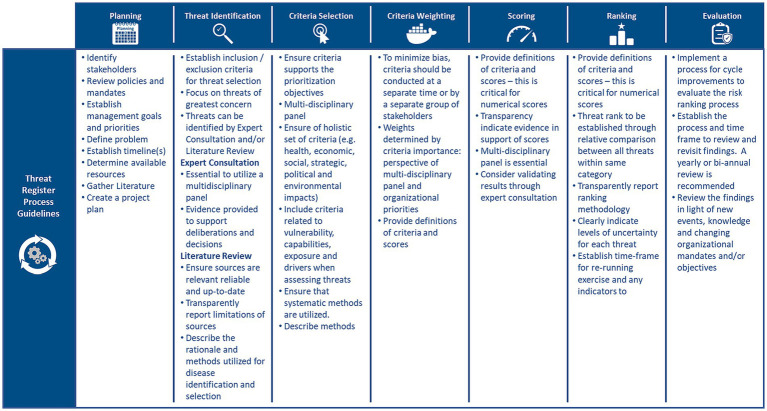
Risk register cycle and the associated principles/guidelines for each step of the threat prioritization exercise (2, 3, 19, 29–31, 33, 34).

Of the 57 articles analyzed, 41 papers specifically mentioned or discussed the design of RRs (3, 21, 29). Five categories emerged from our analysis of RR design: components, process, outputs, methods, and principles. Articles varied in degrees of depth, and specificity. The components that were most consistently included within RRs were hazard identification as well as an analysis of exposure, impacts, vulnerabilities, capabilities, and drivers (19, 30, 31). The elements within each component followed a process of planning (problem formulation, context setting, and scoping), threat identification (e.g., threat inclusion, and exclusion criteria setting), prioritization criteria setting, criteria weighting, scoring, ranking, and evaluation (2, 32, 33). Figure 2 outlines the RR cycle and associated guidelines for each step of the threat prioritization exercise.

The planning stage appeared most frequently in the literature and was highlighted to be integral to RRs as it provides the foundation upon which all other aspects of a RR are built (19, 25, 35). Specifically, in the planning stage, materials (e.g., literature, stakeholders, and policy documents) are gathered to provide insight into the problem(s), and to prepare a project plan (19, 24, 35). Additionally, at this stage, the problem is defined, organizational mandates, objectives, and management goals are identified, and reviewed, the purpose of the prioritization exercise is established, and senior decision-makers are interviewed (19, 36, 37).

**Figure 3:**
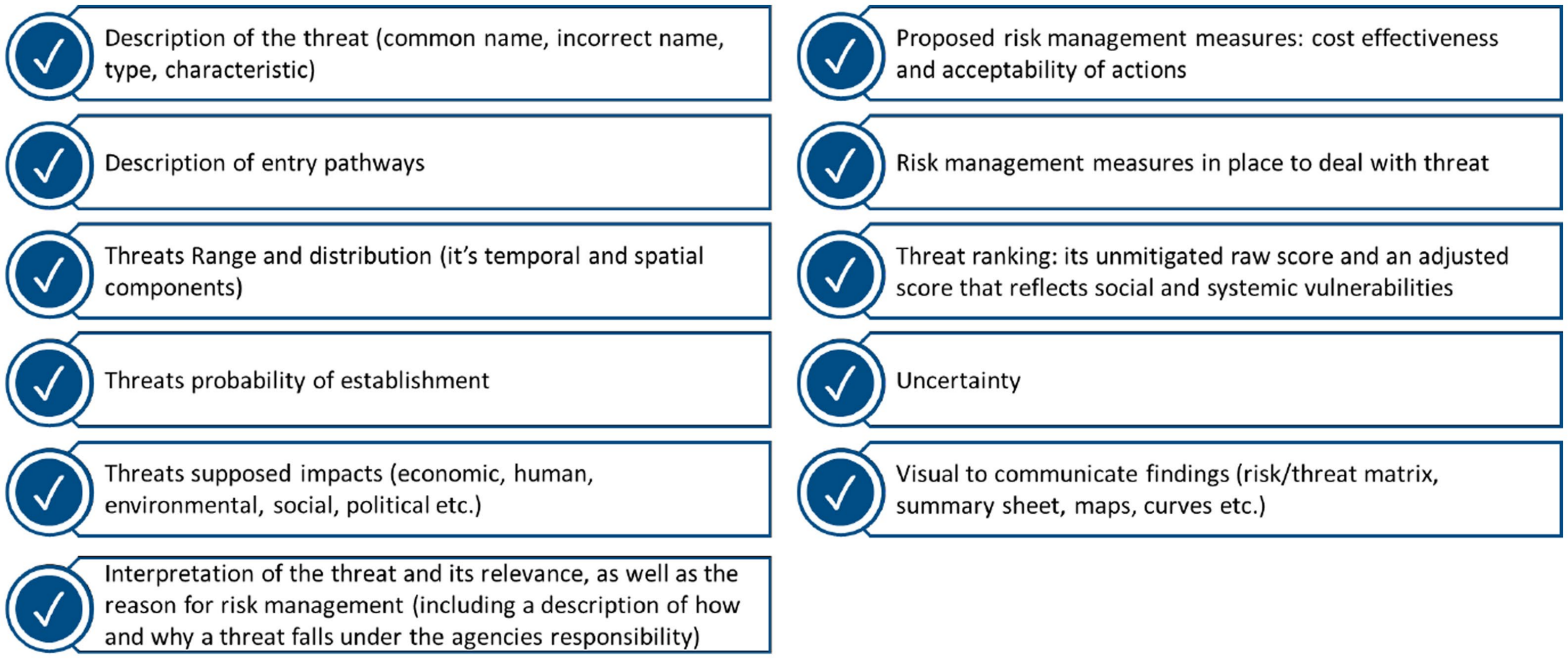
Potential outputs of a risk register (9, 11, 18, 26, 34).

Few articles explicitly mentioned the outputs of a RR- making a thematic analysis problematic. Despite this limitation, we have listed some of the suggested outputs of an RR in Figure 3. It was suggested that for every threat identified, these outputs should be displayed on a summary sheet. Summary sheets should be concise, yet comprehensive- so that decision-makers are provided with all the pertinent information needed to take action without being overburdened with information (9, 18, 26).

### 3.4 Risk register: methods

#### 3.4.1 Methods for stakeholder engagement

**Figure 4:**
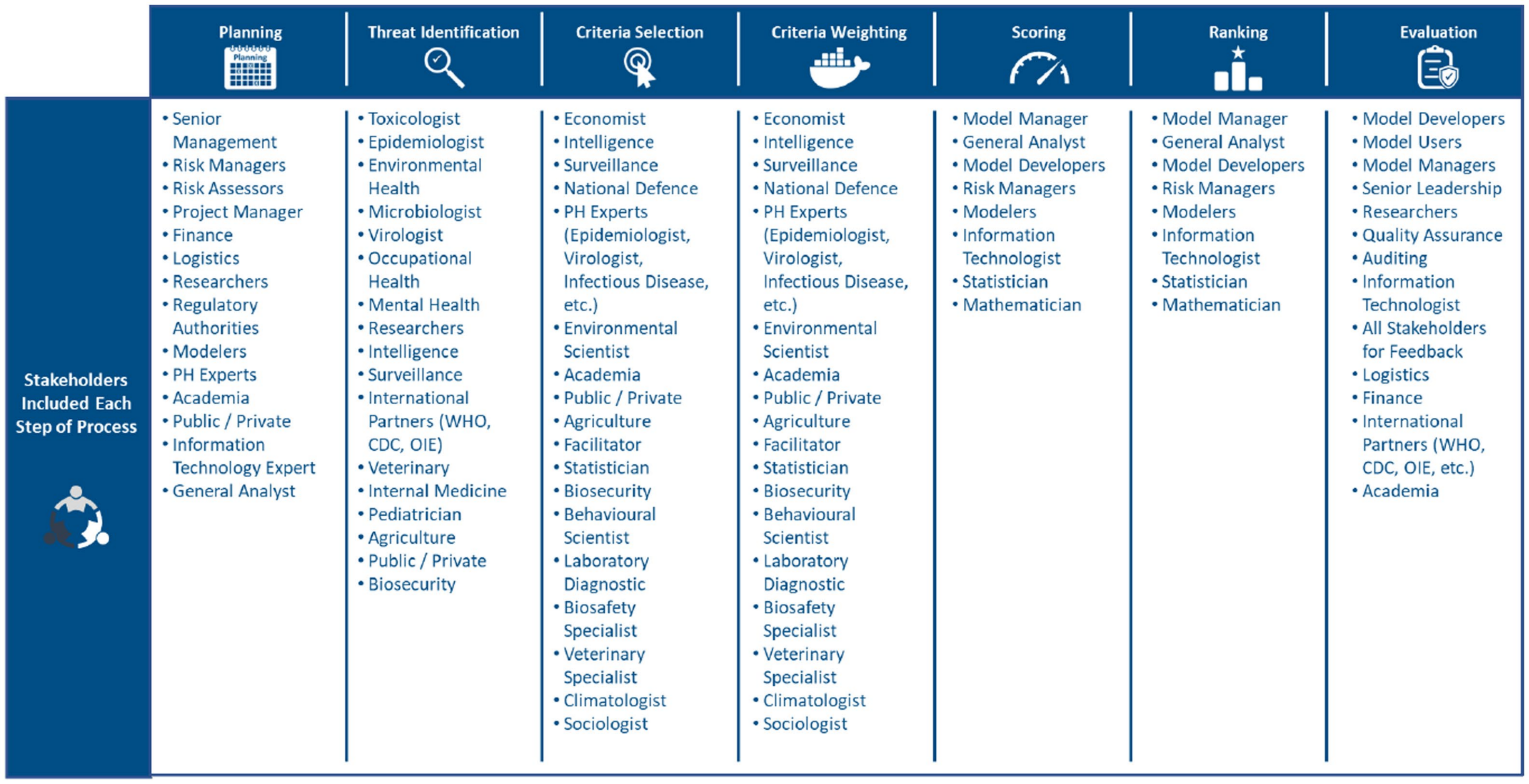
Stakeholders commonly included at each stage of risk prioritization (12, 15, 17, 21, 25, 31, 38, 39).

Most studies utilized subject matter experts to inform the threat prioritization exercise (17, 38, 39). Likewise, studies highlighted that it is essential that a multi-disciplinary and multi-domain panel of experts is selected to inform the threat prioritization exercise (25, 31, 39). Multi-disciplinary and multi-domain panels are needed to respect the multidimensionality of PHTs (12, 15, 21). Figure 4 lists some of the stakeholders included at each step of the threat prioritization exercise. MCDA was the most common method applied to facilitate the incorporation of expert opinion(s), and to guide the threat prioritization process (2, 6, 17). A few articles employed Delphi methods for criteria selection, and weighting. However, the studies that utilized Delphi methods did so in support of, and in conjunction with MCDA, as Delphi methods forced experts to make trade-offs and ultimately reduced bias (2, 5).

#### 3.4.2 Methods for categorizing threats

The two key themes which emerged in our analysis of threat categories were that specific categories were used for similar threats, and broad categories for dissimilar threats (6, 18, 33, 40). Specifically, on the one hand, when threats are of similar origin, they can be categorized by their class characteristics or type (6, 18, 33, 40). For instance, biological PHTs could be categorized into viruses, bacteria, protozoa, and parasites. On the other hand, when disparate threats were being prioritized, generic categories were utilized; for example, malicious, natural, and accidental or vaccine preventable diseases, endemic diseases, rare and imported diseases, hospital related diseases, limited surveillance, and congenital diseases (6, 41).

#### 3.4.3 Methods for identifying, including, and/or excluding threats

Threats can be identified through expert elicitation, literature review or a combination of the two (2, 19, 22, 42). It was asserted that a multi-disciplinary panel was essential if experts were to be elicited, while reliable, and up-to-date sources should inform literature reviews (2, 5, 12). The majority of studies combined both approaches to identify threats: literature reviews were utilized to draft a pre-formulated list, and then this list was provided to experts for feedback and revision (19, 42). MCDA and Delphi methods were regularly implemented to facilitate the process of threat identification (2, 5, 12).

The criteria used to include or exclude a threat were discussed extensively in the literature. First and foremost, inclusion and exclusion criteria should be fit-for-purpose and established deliberatively through expert consultation and specific to the prioritization exercise’s problem, decision, and objective(s) (1, 19, 42). In support of this guidance, most papers ensured that internal and external policies, mandates, and agreements were considered when formulating inclusion and exclusion criteria (i.e., International Health Regulations) (22, 28, 42). In most cases, criteria were selected by drawing upon previous prioritization exercises, and literature reviews, and then finalized through stakeholder deliberations (29, 33, 42, 43).

The majority of articles ensured that threats were included if they were notifiable within internal or external partner reports. Additionally, it was proposed that inclusion criteria should capture atypical threats, those of low probability and high consequence (black swan threats), and threats with the potential for deliberate release (6, 12, 38). Other indicators to include a threat were if PH plays a role when a threat emerges and if a threat tests a PH capability (28, 29, 44). When a large number of threats were being prioritized, it was frequently advised only to include the threats of greatest concern (1, 6, 10). Exposure, vulnerability, likelihood and impact scores obtained from previous prioritization exercises were consistently utilized to determine the threats of greatest concern (6). Threats were excluded if they had minimal to no known impacts, low relevance to the problem, or did not test a capability (1, 29, 44).

#### 3.4.4 Methods and criteria for ranking and prioritizing threats

**Figure 5:**
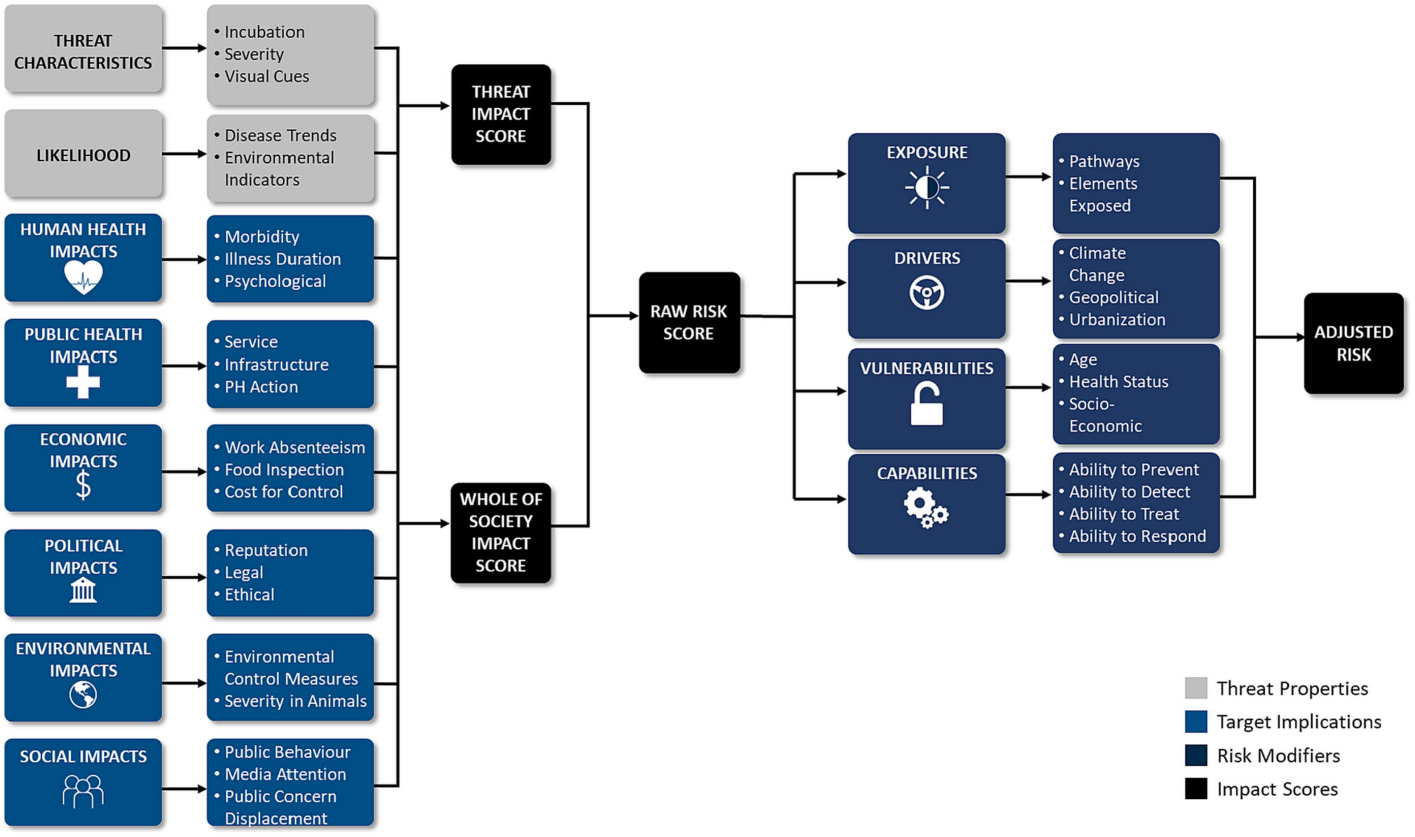
Example flowchart for threat prioritization (2, 3, 19, 21, 29–34).

MCDA and Delphi methods were typically applied to facilitate the selection of criteria for ranking and prioritizing threats (2, 3, 6, 34). MCDA methods were most frequently employed as they support the evaluation of multiple conflicting criteria. A few studies also applied Delphi methods in conjunction with MCDA, as this framework forces stakeholders to make trade-offs- reducing the subjectivity and bias of decisions (2, 5, 45). In most articles, criteria for prioritization were selected by drawing upon previous prioritization exercises and literature reviews, and then finalized through expert feedback (21, 42). The threat prioritization process is outlined in Figure 5.

Although criteria were individualized to the prioritization exercise(s), common criteria emerged, which we categorized into three unique groups: threat properties, threat implications, and risk modifiers. Criteria within these groups included measures of exposure (e.g., inhalation), impacts (e.g., health impacts), capabilities (e.g., ability to respond), vulnerabilities (e.g., health status), likelihood (e.g., disease trend), and drivers (e.g., climate change) (3, 6, 17, 45). Likewise, vulnerability was commonly analyzed when prioritizing threats (22, 45, 46). The most common criteria utilized to prioritize threats were factors related to human impacts, health service impacts, and economic impacts (3, 6, 10, 18, 36). Examples of criteria within each criteria group are presented in Table 1, Table 2, and Table 3. In most cases, criteria were also weighted in terms of importance, and criteria weighting was conducted at a separate time and before ranking threats to reduce bias (2, 9, 34). Las Vegas, Delphi, and MCDA were most readily applied to facilitate the criteria weighting process (2, 9, 12, 34).

**Table 1:** Criteria used based on threat properties.

Aspect of analysis	Criteria examples	
Threat properties		
Threat characteristics	Transmissibility (e.g., speed of transmission, mode of transmission) Incubation Pathogenicity Mutagenicity Distribution in animals Similarity to currently circulating threats Specific animals involved	Visual cues in humans to avoid threat Geographic source of the disease: geographic proximity Severity of symptoms Chronicity of illness or sequelae Threat endemic or exotic Type of threat Human cause versus natural cause
Likelihood	Host movement Threat trend (5 years) Pathways for introduction Potential for transmission Ease of release (accidental or deliberate)	Number of new human cases (5 years) Seasonality of threat Risk of bioterrorism Type of climate threat can tolerate

**Table 2:** Criteria based on threat implications.

Threat implications	
Human impacts	Morbidity and/or mortality (short and long term) Psychological impact Severity of illness (animals and humans) Duration of illness (animals and humans) Reproductive consequences
Public health impacts	Public health action required Health-care service impacts (e.g., laboratory services, mass care etc.) Infrastructure impacts MCM’s required
Economic impacts	Socio-economic burden of threat: on humans and industry Costs resulting from trade and travel restrictions Work absenteeism Cost for control and/or eradication Food inspection programs Heath care and long-term disability costs
Environmental impacts	Impacts of the threat and its control on soil, water, air, landscape and biodiversity Consequences of overstocking, movement controls Impact of medicines or disinfectants Severity of illness in animals
Social impacts	Public/personal concern, disruption, risk perception, attention, and discontent, School absenteeism Changes in behavior Impact of media attention Public service disruptions (water, sanitation, utilities etc.) Population displacement
Political/strategic impacts	Impacts on reputation and credibility Legal implications Ethical obligations

**Table 3:** Criteria based on risk modifiers.

Risk modifiers		
Vulnerabilities	Population information: Age Sex Health-status Ethnicity Population size Population immunity Population density and mobility	Extra planning needs: Communication Evacuation Sheltering Mass care Medical management Plans
Capabilities	Ability to prevent Ability to detect (e.g., effectiveness of national and international surveillance) Ability to diagnose (e.g., effectiveness of testing in animals and humans) Ability to treat (e.g., vaccine) Management needs (e.g., plans, policies or other mobilization required when a threat emerges) Vaccine / antiviral manufacturing time How much is scientifically known Available financial resources, infrastructure, skills for tackling threat Legislative powers, control strategies, contingency plans and disease controls in place to deal with a threat Levels of hygiene in hospitals Healthcare density	
Drivers	Impact of climate-change on threats, vectors, and hosts Threat trends (5 years) – surveillance and intelligence Anti-Microbial Resistance Geopolitical issues Urbanization	
Uncertainty	The quality of scientific evidence used in the assessment Level of expert’s confidence in their opinion(s)	

### 3.5 Risk register timescale and updates

#### 3.5.1 Timescales

Where discussed, it was suggested that timescales should be suitable to the purpose of the threat prioritization exercise, consistent with the processes selected for analysis, long enough to consider the long-term changes (e.g., climate-change), and suitable to capture the time-lag between cascade events (30). A timescale of five-years was most frequently applied and a 10–25-year timescale was suggested for long-term strategic planning (2, 9, 12, 34). It should be noted that the literature also highlighted that when timescales are extended, more uncertainty is introduced, which ultimately reduces the robustness of findings.

#### 3.5.2 Updates and time barriers

In the majority of cases, RRs were revisited, repeated and updated yearly or in line with organizational planning cycles and at regular intervals (6, 42, 47). Our review also observed several triggers to revisit, repeat or update a RR: as new data, information or knowledge arises; as new technologies or interventions are developed or implemented; as the global risk landscape changes; as industry practice(s) that could have on a threat change; and as threat drivers change (1, 2, 25, 44). No themes emerged for time barriers.

## 4 Discussion

### 4.1 Main findings

Our review observed that articles varied in relation to threats prioritized. Specifically, studies within our review covered human, animal and environmental PHTs. Some articles analyzed a single type of PHT (e.g., human), while others examined multiple PHTs. These findings highlighted that PHEM is beginning to acknowledge that a systems approach is necessary to facilitate the analysis of a diverse set of PH threats. In concurrence with literature published in the field of emergency management, our research also discovered that a variety of studies utilized collaborative, multisectoral, and transdisciplinary approaches to prioritize PHTs (2, 12, 42). Specifically, our research demonstrated that criteria should be expanded outside the realm of PH to include economic, environmental, social, and political factors (2, 10, 22). Likewise, an analysis of vulnerability, drivers, exposure, and capabilities is needed to accurately assess the risk posed by PHTs (18, 31, 36, 45).

In the literature searched, two articles were intended for regional and/or local levels of government, however, both studies asserted that their findings could be generalized to other levels of government (24, 43). Although no reasoning was provided for this assertion, our analysis demonstrated that the general structure, principles and methods of an RR are applicable to all levels. As such, we contend that the findings within this paper can likewise be utilized to inform threat prioritization exercises at local, regional, provincial, and national levels. These findings can help support a unified approach in PHEM practice, by defining and establishing a common lexicon and methods for threat identification and assessment. The objective of this paper was to establish BP for the development of RRs within PHEM. The researchers focused on the concepts of risk analysis, prioritization, identification, classification, and characterization. A discussion follows.

### 4.2 Fit-for-purpose

RRs must be clearly articulated from the onset because this defines the problem area and solution space the RR is looking at (19, 25, 27). Specifically, the components of an RR should be fit-for-purpose and integrated within organizational planning cycles and decision-making processes (25, 47). Planning is needed to guarantee that the threat ranking exercise is matched to organizational objectives and developed with established BP and principles (16, 19, 25, 27). For planning to be effectively supported it is critical that senior management is engaged from the onset, so that objectives and priorities are clearly defined (13, 16, 27). PHOs can also seek to leverage internal capacities to establish roles and responsibilities in support of the planning process, as the requisite resources, personnel, and expertise already exist within PHO.

Given that PHEM is currently segmented and disjointed, departments in and outside health need to be fostered and unified (27, 28, 31). Practical steps PHOs can take to support this recommendation include identifying relevant sector inputs, stakeholders, and establishing governance as well as information sharing and knowledge translation processes (27, 28, 39, 47, 48). Communication and cross sectoral engagement are not only supported when methods and terminology are standardized, but it also certifies that efforts are not duplicated, and that resources are effectively and efficiently utilized (27, 28, 30, 37). Lastly, by placing a stronger emphasis on preparedness versus response we can achieve stronger planning, and forward thinking, while moving away from reactive responses to PH emergencies.

### 4.3 Grounded in a system understanding

It is recognized that health issues are multifactorial; as such, a holistic and multidisciplinary approach is needed to support the threat prioritization exercise (30, 34, 41, 42). This understanding is supported when the cumulative knowledge of experts from various disciplines and domains (e.g., environmental scientists, economists, and intelligence) are integrated into the threat identification process (20, 26, 30, 32). Specifically, the utilization of surveillance and intelligence would support the threat prioritization process by revealing threat modifiers such as malicious intent. In sum, the integration of specialized expertise ultimately enhances the validity of findings (26, 30, 34, 41, 42). Thus, to ensure all relevant expertise and knowledge are integrated into threat prioritization exercises it is important to identify and understand the interconnections and linkages between PH and other sectors (27, 30). In doing so, cascading effects, from one sector to another, can also be recognized and addressed.

### 4.4 Transparency, consistency, repeatability, and interoperability

Our review demonstrated that the principles of transparency, consistency and repeatability are critical to ensure that threat prioritization exercises are evidence-based, valid, and auditable (21, 26, 31, 37). To ensure the principles of transparency and consistency are respected, PHOs should ensure that all aspects of threat prioritization exercises are clearly documented (21, 26, 27). Consistency is achieved when methods, processes, and terminology are aligned and unified with all PH partners and at all levels: local, regional, national, and international (21, 26, 30, 37). Standardization also ensures interoperability and can reduce costs and increase the effectiveness of PH services (6, 28). Interoperability is necessary as PHTs are not limited by geography, discipline or domain. As such, the robustness, and quality of threat prioritization exercises is enhanced when interoperability is respected because it enhances coordination, communication, and collaboration with all partners, and this is necessary due to the multidimensionality of PHTs (6, 28). In other words, standardization supports a whole-of-society and whole-of-government approach to managing PHTs, which are BP outlined by the WHO and United Nations Office for Disaster Risk Reduction (6, 11, 49).

### 4.5 Outputs

Our review highlighted that outputs from a RR should strike a balance between comprehensiveness and simplicity so that decision makers are provided with sufficient information to take action, without being overburdened by data (9, 19, 21). However, past experiences have demonstrated that translating risk information to decision-makers can be challenging (27, 30). For instance, before COVID-19 researchers urged PH authorities to prepare for a major influenza pandemic, yet these recommendations were largely disregarded (4). As such, research is needed to enhance current methods for translating risk-information to decision-makers (27, 30). That said, some of these issues can potentially be resolved by ensuring that decision-makers are canvassed and that their information needs, wants, and decision-making processes are identified at the onset (30).

In our review, a few articles specifically discussed the outputs of a RR. However, when the outputs identified were analyzed against the research that did not specifically identify any outputs, the outputs could still be verified. Specifically, the methods, processes, and/or procedures applied in the articles that did not highlight any outputs corresponded with the outputs identified in the literature.

### 4.6 Stakeholder engagement

Our review demonstrated that RRs need to be holistic and grounded in a systems level understanding (27, 30, 49). These findings align with the recommendations proposed by the WHO, who have argued for an all-hazard, whole-of-society, and one-health approach to be applied to PHEM (11). As such, it is imperative that PHOs ensure that various domains, and perspectives are integrated into and throughout the threat prioritization process (12, 24, 27, 30, 49). However, the execution of such an approach is likely to be impeded by the current state of PHEM which is disjointed and segmented. As such, for these principles to be respected it is imperative that a unified PH approach is adopted. For instance, PHOs can create processes for information sharing and collaboration, aim to nurture intra- and inter-organizational relationships, and establish clear governance (27, 30). Likewise, PHOs must also understand and identify the interconnections that exist within the PHT environment as it is composed of numerous threats, with various organizations that have a mandate to manage these threats (27, 30).

### 4.7 Values

When incorporating a diverse set of inputs from various domains and disciplines, stakeholder values and preferences must be managed (18, 20, 27, 30). Our review demonstrated that MCDA and Delphi methods can help manage values by supporting the evaluation of competing inputs and by compelling stakeholders to make trade-offs. Nevertheless, it can be argued that further research is needed to investigate the impact of values more broadly, as values inherently influence beliefs of right and wrong, and good and bad (13, 14, 16, 38). For instance, a utilitarian values perspective would influence participants to maximize the aggregate health outcome of a population, while an egalitarian values perspective, would cause participants to minimize health differences by maximizing the welfare of those who are worst off. In other words, whether inherited, professional or personal, values can modify prioritization results. Consequently, a threat’s priority cannot be measured uniformly when values are not effectively managed. It is possible that addressing and identifying values at the onset of threat prioritization exercises could alleviate some of these issues. However, values are subject to change based on shifts in culture, the political landscape, and/or public desires. As such, threat prioritization exercises should be revisited regularly to certify their relevance. Moreover, values are needed to guarantee that atypical (black swan) threats are not missed (38). Consequently, a further examination of values is needed to identify the BP for managing values so that the consistency, repeatability, accuracy and transparency, of threat prioritization exercises is improved.

### 4.8 Threat identification

The literature reviewed argued that when PHOs are prioritizing a large list of PHTs, the threats with minimal to no known impact and threats of the lowest assumed risk should be excluded (1, 44). As such, it can be argued that this would potentially be a blind spot. However, this issue is nullified since RRs are cyclical and iterative (22, 42). Specifically, RRs should be revisited as the threat landscape evolves and as new knowledge arises (1, 25, 44, 48). Thus, it is vital that RRs are linked to and informed by organizational processes (27, 48). For instance, within a Canadian context, RRs can be linked to and informed by the Global Public Health Intelligence Network as doing so would assure that new knowledge, and threat landscape changes are reflected in threat prioritization exercises.

Integrating processes for PHT monitoring and evaluation is also necessary for threat identification as exposure, and vulnerability to PHTs is constantly shifting in relation to driving forces (2, 10, 26). Specifically, driving forces can either attenuate or intensify exposure and/or vulnerability and as such, the relevance of identified threats will change overtime and by geographic location (26, 30, 34). Consequently, the applicability of PHTs to threat prioritization exercises can only be guaranteed when PHOs reflect upon and assess changes to exposure and vulnerability. Lastly, it is essential that data sources for exposure and vulnerability are developed, identified, and applied to the threat identification process, so that all relevant threats are assessed (30).

### 4.9 Categorizing threats

Studies typically organized dissimilar PHTs into broad categories, while PHTs of similar origin were categorized by their class, characteristics, or type (6, 18, 40, 41). Although broad categories can be utilized to organize PHTs they are also problematic. Specifically, utilizing general categories makes it challenging to compare a broad range of PHTs as different criteria, and inputs are employed to assess and prioritize dissimilar threats. As such, a common denominator is needed to bridge this gap. A promising solution to this issue would be to transition away from the typical risk formula (risk = likelihood x impacts) and move towards measuring risk as a function of hazard, exposure, vulnerability and capacity (11, 30, 45, 49). Likewise, this shift can help PHOs understand why some non-extreme hazards can lead to extreme impacts and disasters, while some extreme events do not (11, 49, 50).

### 4.10 Prioritizing threats

In order to accurately prioritize threats for strategic planning within PHEM, the literature revealed that a holistic set of criteria must be selected, and a systems approach is needed (21, 26, 32, 38).

This can be achieved when PHOs ensure that various domains, perspectives and priorities are represented and integrated into the threat prioritization process (7, 25, 30). Specifically, criteria within PHEM should be broadened to include economic, social, political, environmental, infrastructure, vulnerabilities, drivers, exposure, and capabilities (21, 26, 30, 32, 38).

Assessing capabilities is crucial to threat prioritization as it ensures that resources for the management of threats are accounted for and allows PHOs to differentiate between unmitigated and mitigated risk (31, 45, 46). Specifically, capabilities are negative risk drivers, which negate risk by reducing hazard exposure, and vulnerability. Thus, for PHOs to accurately establish a prioritized RR, capabilities need to be assessed since capabilities can drive risk below an organizations risk-threshold. On the other hand, vulnerability allows PHOs to acknowledge and assess the social and structural determinants of health, which contribute to differential health outcomes, and impacts across society (7, 11, 45, 49, 50). Importantly, vulnerability analysis supports an equity-based approach within PHEM by identifying the needs of at-risk population, and the additional planning requirements for these groups (7, 11, 45, 50). Vulnerability supports an accurate assessment of a threat’s priority by recognizing that risk is not distributed equally amongst society (7, 11, 45, 50). The incorporation of vulnerability also enhances planning efforts by identifying the resources and efforts required to reduce risk (7, 11, 45, 50). For these reasons, it is critical to unify capability-based planning with threat prioritization exercises, so that resources that mitigate risk are accounted for, and that the special planning needs of vulnerable groups are addressed. Our analysis also revealed that criteria cannot be considered uniformly, as the importance of criteria differs. As such, criteria should be weighted (2, 12, 34). In this regard, MCDA was most commonly applied to facilitate the engagement of stakeholders as MCDA allows for conflicting criteria to be included and evaluated. Likewise, Delphi methods were utilized to force stakeholders to make value trade-offs.

Inclusion and exclusion criteria will help address the critical question of scope for RRs. PHOs will, depending on their jurisdictional responsibilities, produce different RRs reflecting the geographic, socio economic and timely variability of the PH threat landscape. A national level RR will be noticeably different from a regional RR (2, 6, 9). Similarly, a RR for a tropical region will focus on threats that are very different from those identified for temperate or cold climates. Environmental threats will be more likely to be considered in regions more frequently exposed to specific natural hazards. Finally, from a socio-economic perspective, poorer countries or regions will have a very different list of priorities compared to richer countries or regions. In other words, it is critical that the context of a RR is clearly established including exposure and vulnerability variables. As such, once again it is essential that RRs are regularly updated as the context and threat landscape are constantly evolving.

### 4.11 Time horizons

Through our literature review, it was highlighted that it is critical to select appropriate time horizons for RRs so that all essential variables are captured within the prioritization process (e.g., threat changes, trends, drivers, as well as the dynamic nature of exposure and vulnerability) (3, 10, 26, 30). Our analysis identified several guidelines to be considered when establishing time horizons for RRs utilized within PHEM. Some examples of these guidelines include ensuring that time horizons are suitable to capture the time lag of cascade events, consistent with the processes selected for analysis (e.g., mutation), and long enough to consider long-term changes (e.g., climate change) (1, 2, 6, 8, 12, 25, 34, 42, 44, 47).

Although these findings suggest that time horizons are inherently fit-for-purpose, our analysis also revealed that a 5-year outlook was most commonly applied to guide the analysis of threats and frame the prioritization exercise (1, 2, 6, 25, 42, 44, 47). In some articles, a longer-term timescale (e.g., 10–25 years) was applied or suggested as such a time horizon can allow decision-makers to consider long-term risk-landscape changes, which allow PHOs to take proactive steps to mitigate anticipated impacts and improve outcomes (9). A longer-term time horizon could ultimately increase the sustainability and effectiveness of decisions. However, extending a time-horizon beyond five years introduces additional uncertainty into the process, which can be counterproductive to the prioritization exercise as well as diminishing the robustness of the exercise’s findings. In practice, it is essential that time horizons are carefully selected so that future changes can be analyzed while also maintaining an acceptable level of uncertainty.

The research suggested that timescales should be consistent with the processes selected for analysis, long enough to consider long-term changes, and suitable to capture time-lag between cascade events (1, 2, 6, 25, 42, 44, 47). However, our analysis revealed an issue with this finding. Specifically, if analyzing a broad range of threats, the timescales needed to capture changes will differ (e.g., bacteria vs. virus etc.). As a result, on the one hand, a short timescale may inflate the risk of rapidly changing threats and may potentially miss more significant long-term risks, while on the other hand, a long timescale would do the opposite. Consequently, timescales need to be selected judiciously and the limits of each timescale need to be understood. Further research is needed to develop our understanding of timescales, so that PHOs can determine when one timescale should be used over another. This research is also an opportunity, as identifying timescales for each threat and criteria under examination, could support threat categorization, and comparison, as well as increase the accuracy of assessments.

### 4.12 Revising RRs

The literature highlighted that it is imperative that RRs are viewed as an iterative process, and as such, they should be regularly revisited and updated at consistent intervals. Given these guidelines, the literature demonstrated that it is essential that updates for a RR are established within organizational planning cycles. Specifically, RRs should be revisited, repeated, and updated at least once per year. RRs should also be updated when certain triggers arise; effective planning is needed to map triggers to guarantee that triggers are promptly identified. To do so, internal and external capacities can be leveraged. For instance, within a Canadian context, the Global Public Health Intelligence Network could be utilized to inform threat prioritization exercises by identifying changes and patterns in the threat environment. Likewise, since PHTs are influenced by factors outside of health, it is vital that information from other domains and disciplines are also considered (e.g., business, intelligence, technology, environment, etc.). For these reasons, it is essential that sufficient resources are allocated to manage RRs, so that they remain relevant to the threat environment, to an organization’s needs, and for strategic planning.

## 5 Strengths, limitations, and further research

Our literature review provided several benefits for the practice of PHEM. One key benefit of our research is that it clearly identified the design and process for threat prioritization exercises within PHEM. Additionally, this review identified criteria that can be utilized to inform threat prioritization exercises and enhance the robustness of methods. Likewise, our review also established a variety of BP and principles that, when applied, streamline and align processes across PHOs and ensure consistency of methods. This review also harmonizes communication and collaboration between PHEM partners by establishing methods and key terminology which ultimately support interoperability. An important strength of this review is that it developed PH practice by integrating knowledge from emergency management to PH. Lastly, this review provided PHEM practice with a scalable approach for the management of PHTs that can be used at various levels including whole-of-government or whole-of-society.

A limitation identified in this review was the scarcity of research on threat prioritization in PHEM, where emergency management concepts and knowledge were rarely incorporated. Grey literature was not included in the primary search strategy; however, it may have supplemented some of the findings given the scarcity of the literature. This limitation may have been compounded by the difficulty in conducting a comprehensive search of literature on risk registers due to the number of potential synonyms for both ‘risks’ (e.g., threats, hazards, or specific risk groups) and ‘registers’ (e.g., registry, database, catalogue). One other limitation of this study given the focus of the analysis on disparate threats in PHEM, providing general recommendations or conclusions can prove to be difficult as specific recommendations or limitations may not be fully represented within the thematic analysis conducted. We understand that by limiting our search to 2010, we may have missed some relevant articles, but we determined that this timeline was suitable given the publication date of the ISO 31000.

A cross-disciplinary review of the literature could have uncovered further insights; however, such a search would not have been feasible given the time and resources available. Further research is needed to understand how drivers (e.g., climate change) modify risk overtime including, the considerations for cascade effects, as threats can trigger a sequence of consequences with significant magnitude which can ultimately influence risk. Owing to the heterogenous nature of PHTs, it is essential to investigate criteria and metrics that support the comparison of threats across various categories. Lastly, the exploration of malicious threats and their considerations must be comprehensively explored (51, 52). Research is required to identify security considerations related to information sharing related to malicious threats.

Geographic scope needs to be considered since a RR with a local scope will not register the same threats as a national or sub-national RR. At the same time, multiple coexisting and converging RRs that cover various scopes are needed to efficiently manage PHTs at all levels. Interoperability is also essential for coordinated efforts and to ensure efforts are not duplicated (51). Thus, it can be argued that dissimilar threats should be stationed within macro level RRs, while local or specialty RRs might be needed in more technical contexts.

## 6 Conclusion

Through this review, we identified the BP and principles for the development of RRs within PHEM. These results highlighted the importance of recognizing the multidimensionality of threats, the need for multi-sectoral inputs, and vulnerability analyses. Ultimately, adopting RRs within PHEM can ensure that resources are efficiently and effectively allocated to the most pressing problems, ultimately mitigating, reducing or preventing the economic and social costs of the emergence or re-emergence of PHTs.

## Data availability statement

The original contributions presented in the study are included in the article/Supplementary material, further inquiries can be directed to the corresponding author.

## Author contributions

DK and JZ carried out the literature review including collection of articles, screening of articles, data extract, data analysis, and synthesis of results. DK wrote the first draft of the manuscript. JZ, LT, ML, and BH revised the manuscript, read, and suggested a subsequent round of revisions for the manuscript. DK completed the final revision of the manuscript. All authors contributed to manuscript revisions, read, and approved the submission of the manuscript.
