# Supplementary material for: A rapid review of best practices in the development of risk registers for public health emergency management
Source: Front Public Health. 2023 Nov 30;11:1200438. doi: 10.3389/fpubh.2023.1200438 (PMC10720617; doi:10.3389/fpubh.2023.1200438)
Supplement: Supplementary file 1 [file Table_1.docx]

**Appendix A**

Appendix A: Search strategy and keywords utilized to conduct the literature review.

| **#** | **Searches** | **Results** |
| --- | --- | --- |
| 1 | exp public health/ | 8412099 |
| 2 | ("public health" or "population health" or public safety or (communit* adj2 health*)).tw,kf. | 364017 |
| 3 | (health* adj2 (system* or deliver* or model* or practice*)).tw,kf. | 221664 |
| 4 | or/1-3 [public health] | 8657708 |
| 5 | *disasters/ or *emergencies/ or exp *natural disasters/ or *mass casualty incidents/ or *disaster victims/ | 40473 |
| 6 | exp *disease outbreaks/ | 90242 |
| 7 | *biological warfare agents/ | 236 |
| 8 | (health adj4 (risk* or threat* or hazard*)).ti,kf. | 20829 |
| 9 | (epidemic* or pandemic* or outbreak*).ti,kf. | 129085 |
| 10 | (disaster* or calamit* or catastrophe* or state of emergency or cbrne or nrbc or cbrn or bioterror* or biowar* or (bio* adj (war* or terror*)) or ((large scale* or public) adj3 (emergency or emergencies or evacuation*)) or ((event* or incident or incidents or accident or accidents) adj3 (chemical* or toxic* or biologic* or aviation or extreme weather or extreme water)) or ((mass or large* number* or high* number* or major or multiple) adj3 (casualties or injured or wounded or victims or fatalities or deaths or loss of life or evacuat* or evacuees))).ti,kf. | 23796 |
| 11 | ((major or large* or enormous* or massive* or extensive damage* or traged* or tragic* or devastation or evacuat* or evacuee* or destruct* or ravage*) adj6 (fire* or wildfire* or avalanche* or flood* or storm* or hurricane* or tornado* or earthquake* or landslide* or tsunami* or volcan* or aircrash* or crash* or rail* or derail* or explosion* or shipwreck* or mining accident* or power outage*)).ti,kf. | 1081 |
| 12 | ((extensive damage* or traged* or tragic* or devastat* or evacuat* or evacuee* or destruct* or ravage*) adj6 (extreme weather or exposure* or exposed or contamina* or poison* or pollutant* or biohazard* or hazmat* or bioterror* or biowar* or terror* or attack*)).ti,kf. | 218 |
| 13 | or/5-12 [disaster + emergencies] | 241216 |
| 14 | exp *risk assessment/mt or exp *disaster planning/mt | 20432 |
| 15 | ((impact* or risk or threat*) adj3 (assess* or evaluat* or register*)).ti,kf. | 54291 |
| 16 | ((disaster* or pandemic* or outbreak* or emergency) adj (prepar* or management or plan or plans or planned or planning or readiness)).ti,kf. | 5789 |
| 17 | or/14-16 [risk assessments + disaster planning] | 77327 |
| 18 | 4 and 13 and 17 | 7849 |
| 19 | exp *guideline/ or *guidelines as topic/ or exp *practice guideline/ or *practice guidelines as topic/ or *health planning guidelines/ | 59002 |
| 20 | (guideline* or best practice* or framework* or tool?kit or recommend* or design*).ti,kf. | 375762 |
| 21 | (method* or priorit* or characterization* or characterisation* or management*).ti,kf. | 1383611 |
| 22 | or/19-21 [guidelines + best practices] | 1743022 |
| 23 | 18 and 22 | 1446 |
| 24 | limit 23 to yr="2010-Current" | 1096 |
| 25 | exp australia/ or austria/ or exp baltic states/ or exp belgium/ or exp canada/ or chile/ or colombia/ or czech republic/ or exp "scandinavian and nordic countries"/ or exp france/ or exp germany/ or greece/ or hungary/ or exp ireland/ or israel/ or exp italy/ or exp japan/ or exp republic of korea/ or luxembourg/ or mexico/ or exp netherlands/ or exp new zealand/ or poland/ or exp portugal/ or slovakia/ or slovenia/ or exp spain/ or exp switzerland/ or turkey/ or exp united kingdom/ or exp united states/ or (australia* or new south wales or queensland or tasmania or victoria or sydney or melbourne or brisbane or adelaide or austria* or vienna or viennese* or belgium* or belgian* or brussels or flemish* or canad* or ottawa* or british columbia* or colombie britannique* or vancouver* or alberta* or edmonton* or calgar* or saskatchewan* or regina* or saskatoon* or manitoba* or winnipeg* or ontari* or toronto* or quebec* or montreal* or new brunswick* or nouveau brunswick* or fredericton* or nova scotia* or nouvelle ecosse* or halifax* or haligonian* or prince edward island* or ile du prince edouard* or pei or charlottetown* or newfoundland* or terre neuve* or labrador* or nfld or yukon* or whitehorse* or northwest territor* or territoires du nord ouest* or nwt or yellowknife* or nunavut* or iqaluit* or chile* or santiago or colombia* or bogota or czech* or prague or denmark* or danish or dane* or faroe* or copenhagen or estonia* or tallinn or finland* or finnish* or helsinki* or france* or french* or paris* or marseille or lyon or lille or nice or toulouse or bordeaux or german* or deutschland* or berlin* or hamburg or munich or cologne or frankfurt or stuttgart or dusseldorf or greece* or hellenic* or greek* or athens or macedonia* or hungary* or hungarian* or budapest or iceland* or reykjavik or ireland* or irish* or dublin* or israel* or jerusalem or tel aviv or italy or italian* or rome or milan or naples or turin or sicily or japan* or tokyo or yokohama or osaka or nagoya or sapporo or kobe or kyoto or korea* or seoul or busan or daegu or daejeon or gwangju or incheon or ulsan or latvia* or riga or lithuania* or vilnius or luxembourg* or netherland* or holland* or dutch* or amsterdam or rotterdam or hague or new zealand* or aotearoa or wellington or auckland or maori or mexic* or norway* or norwegian* or oslo or poland* or polish or warsaw or krakow or wroclaw or lodz or portug* or lisbon or slovak* or bratislava or slovenia* or slovene* or ljubljana or spain* or spanish* or spaniard* or madrid or barcelona or catalonia* or valencia* or seville or zaragoza or malaga or basque or scandinavia* or sweden or swedish or swede* or stockholm or switzerland* or swiss* or zurich or geneva or bern or turkey or turkish or istanbul or constantinople or britain* or british* or united kingdom* or scotland* or scottish or wales* or welsh or england* or belfast or london or manchester or glasgow or birmingham or leeds or bradford or liverpool or alabama* or alaska* or arizona* or arkansas* or california* or colorado* or connecticut* or delaware* or florida* or georgia* or hawaii* or idaho* or illinois* or indiana* or iowa* or kansas* or kentucky* or louisiana* or maine* or maryland* or massachusetts* or michigan* or minnesota* or mississippi* or missouri* or montana* or nebraska* or nevada* or new hampshire* or new jersey* or new mexico* or new york* or north carolina* or north dakota* or ohio* or oklahoma* or oregon* or pennsylvania* or rhode island* or south carolina* or south dakota* or tennessee* or texas* or utah* or vermont* or virginia* or washington* or west virginia* or wisconsin* or wyoming* or montgomery* or juneau* or anchorage* or phoenix* or little rock* or sacramento* or los angeles* or san diego* or san francisco* or denver* or hartford* or dover* or tallahassee* or miami* or orlando* or atlanta* or honolulu* or boise* or springfield* or chicago* or des moines* or topeka* or frankfort* or baton rouge* or new orleans* or augusta* or annapolis* or boston* or lansing* or detroit* or st?paul* or jackson* or jefferson city* or helena* or lincoln* or carson city* or reno* or las vegas* or concord* or trenton* or santa fe* or albany* or raleigh* or bismarck* or columbus* or oklahoma city* or salem* or harrisburg* or providence* or columbia* or peirre* or nashville* or austin* or dallas* or salt lake city* or montpelier* or richmond* or olympia* or seattle* or charleston* or madison* or cheyenne* or district of columbia* or usa or united states or ("U.S." adj8 (NASA or EPA or "environmental protection agency" or FEMA or "federal emergency management agency" or HHS or "health and human services" or HUD or DOE or DOD or DOA or DOC)) or "US Department of").tw,kf. | 4796820 |
| 26 | "Organisation for Economic Co-Operation and Development"/ | 373 |
| 27 | (OECD? or ((Organisation or Organization) adj3 ("Economic Co-operation and Development*" or "Economic Cooperation and Development*"))).tw,kf. | 5683 |
| 28 | or/25-27 [OECD countries] | 4800548 |
| 29 | 23 and 28 [OECD countries no date limit] | 557 |
| 30 | 24 and 28 [OECD countries with date limit] | 424 |

**Appendix B**

- How should a RR be designed?
- How can threats be identified?
- What criteria can be used to include or exclude risks/threats from a RR?
- What methods can be used for ranking/prioritizing threats?
- How can risks/threats be categorized?
- What best practices or principles have been established?
